# Development and evaluation of a novel, real time mobile telesonography system in management of patients with abdominal trauma: study protocol

**DOI:** 10.1186/1471-227X-12-19

**Published:** 2012-12-18

**Authors:** Chinwe Ogedegbe, Herman Morchel, Vikki Hazelwood, William F Chaplin, Joseph Feldman

**Affiliations:** 1 Emergency Trauma Department, Hackensack University Medical Center, 30 prospect Avenue, Hackensack, NJ, 07601, USA; 2 Department of Psychology, St. John’s University, 8000 Utopia Parkway, Jamaica, NY, 11439, USA

**Keywords:** Telesonography, Abdominal trauma, Patients, Pre-hospital, Novel, Real-time, Mobile

## Abstract

**Background:**

Despite the use of e-FAST in management of patients with abdominal trauma, its utility in prehospital setting is not widely adopted. The goal of this study is to develop a novel portable telesonography (TS) system and evaluate the comparability of the quality of images obtained via this system among healthy volunteers who undergo e-FAST abdominal examination in a moving ambulance and at the ED. We hypothesize that: (1) real-time ultrasound images of acute trauma patients in the pre-hospital setting can be obtained and transmitted to the ED via the novel TS system; and (2) Ultrasound images transmitted to the hospital from the real-time TS system will be comparable in quality to those obtained in the ED.

**Methods:**

Study participants are three healthy volunteers (one each with normal, overweight and obese BMI category). The ultrasound images will be obtained by two ultrasound-trained physicians The TS is a portable sonogram (by Sonosite) interfaced with a portable broadcast unit (by Live-U). Two UTPs will conduct e-FAST examinations on healthy volunteers in moving ambulances and transmit the images via cellular network to the hospital server, where they are stored. Upon arrival in the ED, the same UTPs will obtain another set of images from the volunteers, which are then compared to those obtained in the moving ambulances by another set of blinded UTPs (evaluators) using a validated image quality scale, the Questionnaire for User Interaction Satisfaction (QUIS).

**Discussion:**

Findings from this study will provide needed data on the validity of the novel TS in transmitting live images from moving ambulances to images obtained in the ED thus providing opportunity to facilitate medical care of a patient located in a remote or austere setting.

## Background

Acute trauma is a leading cause of morbidity and mortality in the United States [1-3]. Care of patients with acute trauma costs the US government an estimated 27 billion dollars per year [1-3]. Reasons for the increased cost of care include the complexity of care, which in turn increases exponentially with increased lag time between occurrence of trauma and provision of definitive care by the trauma team at the hospital. For example, diagnosis and management of patients with acute trauma often require accurate pre-hospital diagnosis; rapid transmission of diagnostic and clinical data by paramedics to the emergency department (ED); and provision of definitive care by a multidisciplinary trauma team including the ED physicians, radiologists, and trauma surgeons. The first hour of trauma, often termed the ‘golden hour’, is the most crucial predictor of mortality in patients with acute trauma [4-7]. Published studies indicate that the major barrier to reduction of time to surgery in most trauma patients is the lack of adequate diagnostic capability of the ED physician [8-10].

Point-of-care ultrasound has been used by non-radiologists of various specialties including emergency medicine, critical care medicine, and surgery at the bedside to help answer specific point-of-care questions that may affect immediate patient care [11-18]. The widely adopted Focused Assessment with Sonography for Trauma (FAST) has reduced the time-to-surgery by training of ED physicians to accurately diagnose acute abdominal injuries, often a common cause of death in patients who present to the ED with acute trauma [11-18]. Although FAST is now widely employed in most tertiary EDs across the country, its ability to reduce the time-to- diagnosis is still significantly limited [19-22]. This is largely due to the paucity of specialists trained in the use of FAST and the lack of trauma expertise in community-based hospital EDs, where majority of patients with acute trauma receive care. Especially notable is the inefficiency in the use of the ‘downtime’ during which patients are transported from the pre-hospital setting to the ED. This ‘downtime’ provides an opportunity to reduce the time-to-diagnosis during transportation from the prehospital setting by paramedics to the ED.

Recent technological advances in broadband and satellite communications systems, the increasing role of telemedicine, and the availability of portable ultrasound scanners provide a unique opportunity to address this problem. A first responder provider such as paramedic may perform a FAST exam with the remote guidance from an experienced expert or UTP, to furnish crucial information during the ‘golden hour’. This technology will provide the opportunity to employ ‘real time transmission’ of ultrasound images (telesonography) from the pre-hospital setting, and during transportation to the ED. The inclusion of two-way voice, and one-way video communications from the first responder (paramedic) to the ED physician may further enhance the first responder’s abilities to accurately and efficiently evaluate the patient. Although the feasibility of telesonography (TS) is proven in a couple of studies, more technical development and clinical data are warranted [23,24]. To date, we are not aware of any published data on the development and use of this approach for patients with blunt abdominal trauma.

The goal of this study is to develop a novel telesonography (TS) system and evaluate the comparability of the quality of images obtained via this system among healthy volunteers who undergo e-FAST abdominal examination in a moving ambulance and at the ED. We hypothesize that: (1) real-time ultrasound images of acute trauma patients in the pre-hospital setting can be obtained and transmitted to the ED via the novel TS system; and (2) Ultrasound images transmitted to the hospital from the real-time TS system will be comparable in quality to those obtained in the ED.

## Methods

### Overview of study design

The design of this proposed study is patterned after the work of Sibert et al. conducted in similar settings [25]. Thus the study will be conducted in two phases – phase 1 (development phase) and phase 2 (evaluation phase). During phase 1, we will develop and test the TS system by interfacing a portable ultrasound and a broadcast unit. For this purpose, we will determine the capability of the TS system to transmit quality images from a pre-hospital setting to the ED. During phase 2, we will evaluate the usability of the novel TS system with two-way voice and one-way video communications capability and then compare the quality of the ultrasound images obtained real-time, from healthy volunteers in a moving ambulance via the developed TS system to those obtained in the ED; thus assessing the performance characteristics of the TS system. For this purpose, two ultrasound-trained physicians (UTPs) will conduct e-FAST examinations on 3 healthy volunteers in moving ambulances and upon arrival to the ED. Upon completion of the eFAST examination, the images obtained in the moving ambulances and in the ED will then be compared to each other by another set of UTPs (evaluators) who are blinded to the study objectives. The quality of the images will be compared using a validated image quality scale, the Questionnaire for User Interaction Satisfaction (QUIS) - a reliable and valid tool developed by a team of researchers in the Human-Computer Interaction Laboratory at the University of Maryland, College Park, [25,26] and designed to assess users' satisfaction with specific aspects of the human-computer interface. In its current version, QUIS 7.0, contains a demographic questionnaire, a measure of overall system satisfaction along six domains, and hierarchically organized items of nine specific interface factors (screen factors, terminology and system feedback, learning factors, system capabilities, multimedia, for example). Each domain evaluates the users' overall satisfaction with that facet of the interface, as well as the factors that make up that facet, on a 9-point scale.

### Study setting and participants

Based on a prior study conducted by Sibert and colleagues (2007), which included seven raters of a similar sonogram system, which had enough power to demonstrate reliability. The power analyses based on the results of the Sibert study the effect size was .67 therefore a power of .80 with a level significance set at .05 we need 16 raters. In this study we are erring on the conservative side and plan to include a total 20 raters.

The study will be conducted in the adult ED of Hackensack University Medical Center (HUMC) and on HUMC ambulances. The study participants will be three healthy adult volunteers of various ages (18 to 65 years of age), body mass index (normal; overweight and obese), and gender. All study volunteers will be identified via the HUMC volunteer office. Twenty UTPs (evaluators) including radiologists, emergency department (ED) physicians, and trauma surgeons with experience in performing e-FAST examinations will also be recruited into the study. Pregnant women, Non-English speaking subjects and those with cognitive disabilities that would impair their ability to understand the informed consent process will be excluded from the study. The study protocol was approved by the Institutional Review Board of HUMC and all study participants are required to provide informed consent.

### Recruitment and informed consent

Potential study participants will be recruited from the volunteer office of HUMC. Protocol of the study will be explained by a research assistant (RA). Volunteers who agree to participate will then be asked to provide written informed consent. Participants will receive compensation of $20 for their time and travel. The UTPs (evaluators) will be randomly selected from the hospital’s roster of UTPs and approached by one of the investigators to participate in the study. All UTPs will also be required to provide written informed consent.

### Characteristics of the TS system

The system utilizes a patented technology (US Patent No: 7,948,933) developed for the broadcast industry by LiveU Corporation, Hackensack, New Jersey to take the video output of a standard medical ultrasound device and transmit the image in real time to a hospital or any other location. The video stream from the ultrasound device is transmitted in its entirety so the frame rate is preserved and decoded on the receiving end. The technology was developed by LiveU Corporation for high definition media/broadcast images and is being used by the major broadcast networks. The system was adapted for use with medical ultrasound in collaboration with Hackensack University Medical Center. The system utilizes proprietary implementations of video encoding/compression standard H.264, which provide adaptive bit rate, adaptive and predictive forward error correction, and error recovery mechanisms. The transmission system has a built in proprietary passive antenna to support multiple signals including 3G and 4G LTE. Multiple modular wireless communications links are employed which can include any cellular system including the latest technology such as LTE, WiMax, HSPA+, and the system is backward compatible with existing technology such as CDMA, FDMA, TDMA, WCDMA, WIFI. Satellite links such as BGAN and VSAT are also supported. Cellular modems or other communications devices for the desired link plug into LiveU system to provide connectivity. The desired bandwidth is achieved by tagging the digital words representing the ultrasound video images with identifiers separating and transmitting them over the multiple communications links, and then re-assembling the digital video stream at the receiving end to re-create the images. Using multiple independent communications channels to transmit the separated digital words multiplies the available bandwidth. The multiple lines are monitored in real-time (both delay, packet loss and bandwidth) and packets are transmitted according to their priority.

### Study procedure

**Figure 1:**
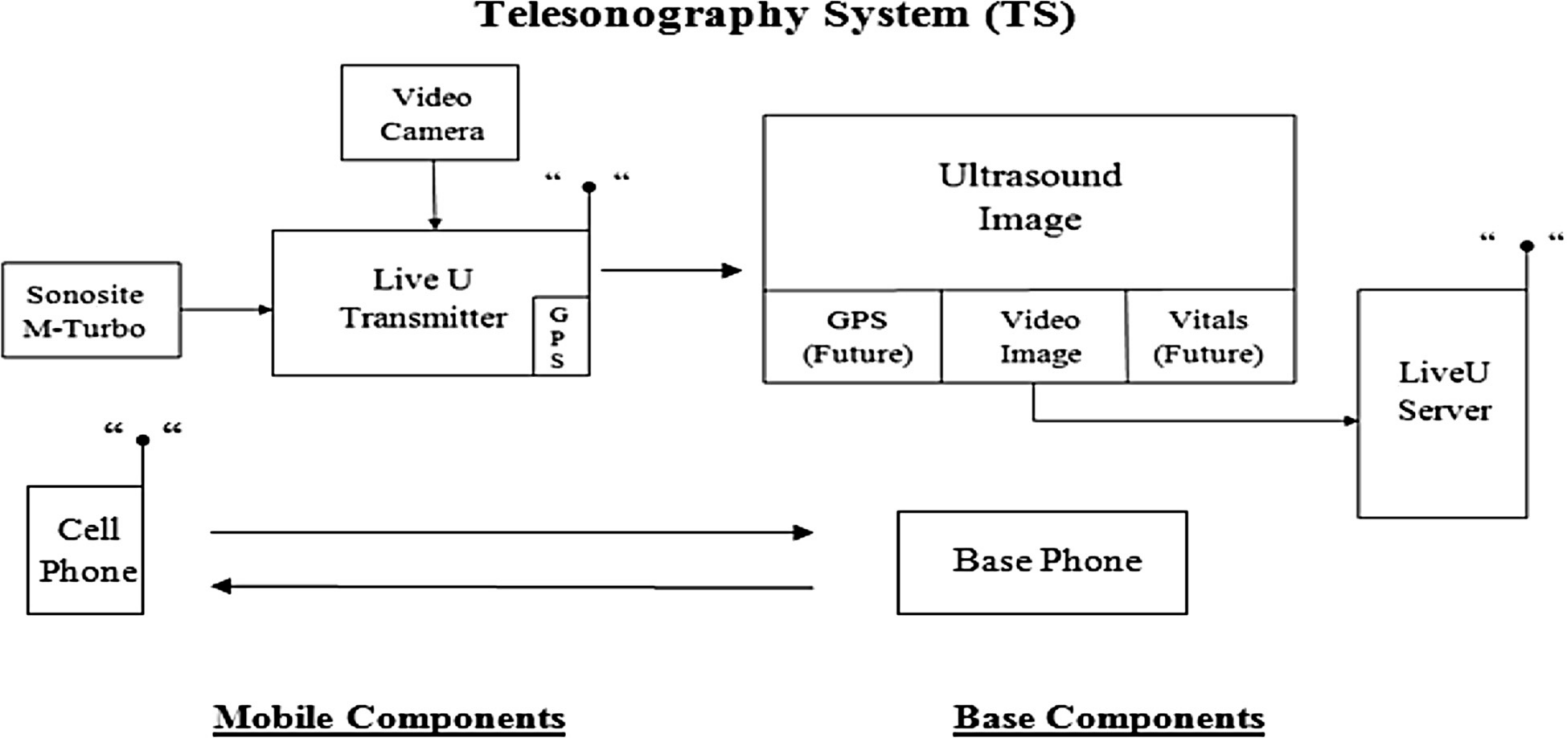
Overview of the Telesonography System.

The study procedure is planned in four sequential steps: in Step I, a novel recently developed portable ultrasound machine interfaced with a broadcast unit, (TS system) with real time image transmission capabilities will be used to obtain images from healthy volunteers. This TS system transmits over commercial cellular networks, utilizing multiple channels simultaneously. The audio data will be transmitted over one cellular channel, while the video data is multiplexed and transmitted over multiple cellular channels. Advanced digital signal processing is utilized to perform video compression, compensate for signal dropouts and manage cellular data links. A monitor that is installed at the physician workstation receives images from the portable TS system. There is an association between the image frame rate and image quality, such that the lower the frame rate, the better the quality of the image. This system allows the transmission of images from the mobile component in the ambulance to the base component in the ED (see Overview of Telesonography System in Figure 1).

In Step 2, healthy volunteers are consented and taken to the ambulance. In Step 3, volunteer subjects will ride on HUMC ambulance along with a UTP who then performs an the extended-Focused Assessment with Sonography in Trauma (e-FAST) examination while the ambulance is in transit using the Sonosite M-Turbo portable ultrasound system (Bothell, WA) connected to a proprietary telebroadcasting system provided by LiveU (Paramus, NJ). The Sonosite M- Turbo portable ultrasound system by (Sonosite Inc.) will be equipped with a C 15/4-2 m Hz transducer for the e-FAST abdominal exams. Images from the e-FAST examination will be acquired by the UTP and transmitted via the real-time TS system to the home base, from where they are then recorded on the hospital servers. Simultaneous to the video images being sent, the UTP will communicate via 2-way audio with the physician in the ED base station at the hospital. The e-FAST examination is conducted on three areas of the body the pelvis, abdomen and chest. Upon arrival of the ambulance at the ED, in Step 3, the same UTP performs a second e-FAST examination on the same healthy volunteer while stationary at the ED. These views will again be recorded in sepa-rate files on the hospital servers. Finally, in Step 4, the images of the e-FAST examinations obtained from both the moving ambulance and at the ED, will be compared by twenty UTPs (evaluators) who are blinded to the study. Comparison of the images will be evaluated with the well-validated QUIS instrument. For this purpose, each UTP evaluator will rate the ambulance using the QUIS instrument.

### Data collection

#### Demographic data

Upon entry into the study, volunteers are asked to complete a demographic questionnaire that asks about their age, ethnic and racial category, gender, exact height, weight, and identification of any chronic medical conditions, if any. They are then weighed and their height measured using standardized scales. These measurements are used to calculate their body mass index (BMI).

#### Sonographic data

Six pre-determined views of e-FAST images of different regions of the body (Left and Right chest, hepatorenal, splenorenal, suprapubic, cardiac—parasternal long axis or subxiphoid) are obtained in the ambulance, and transmitted via the telesonography system to the hospital for storage in the hospital servers. The same views are obtained in the Emergency Department by the UTP upon arrival of the ambulance at the hospital. The images are also stored on the servers.

#### Usability data

The data on usability of the TS and its images (both those obtained on the moving ambulance and in the ED) are collected via completion of the QUIS by 20 UTP evaluators, who are blinded to the study. For this purpose, the sets of images obtained (pre-hospital and in the emergency department) are reviewed for quality along the following dimensions: a) Total image quality, defined as an overall assessment encompassing contrast of solid and fluid-filled structures, and absence of noise; b) Image resolution, defined as the sharpness and crispness of the image and a lack of haziness/blurriness; and c) Image detail, defined as the clarity of organ outlines and ease with which boundaries of structures are seen and how well they are defined [27-29]. The blinded UTP evaluators rank their impression of the images along the dimensions listed above using a 9-point Likert scale with 1 being the worst and 9, the best image resolution. In addition to QUIS, the evaluators are also asked to complete the following tasks: 1) compare the images on the ambulances with that taken in the ED, and then rate them as equal, inferior or superior; 2) identify any pathology in the images as well as list the major visible structures; and 3) rate whether the image being viewed is either suboptimal for review or contains image artifacts other than expected from ultrasound. Evaluators then enter their responses on the QUIS data form, from where the data are entered into a computerized database.

### Data analysis management

The ratings in each condition will be described using means and standard deviations computed across the 20 UTP evaluators. Additionally correlations among the 3 image ratings in the separate conditions across the 20 raters will be reported. We do not anticipate that there will be much, if any, missing data so the primary analysis will be repeated measures MANOVA. Specifically, images will be available on three different healthy volunteers (study participants) at six separate body sites (both sides of the chest, abdomen and pelvis) obtained under two conditions (moving ambulance and still images in the ED). Each image will be rated by 20 evaluators on three scales (Quality, Resolution, and Detail). Thus we will have 3 × 6 × 2 × 3 design with 20 raters in each cell. The primary analysis will be to compare the ratings of the 20 raters between the two conditions on the 3(volunteers) × six (body sites) × 3 (ratings). The N for each comparison will be 20 (evaluators). The primary hypotheses test will be the main effect for condition (moving versus still) and the interactions involving condition. If significant interactions are obtained it would suggest that there are differences between moving and still images as a function of body site, individual being imaged, and/or type of image characteristic (Quality, Resolution, Detail). Any such effects will be followed up using Bonferroni adjusted post-hoc comparisons. Prior to conducting the above analyses, a generalizability analysis of the 20 raters will be conducted to establish the degree of rating consistency (“reliability”) among the 20 raters across the facets of the study (individuals × body sites × condition × rating) will be assessed and indexed using coefficient alpha. Any raters who show poor overall agreement may be excluded form the final analysis.

### Expected results

The expected results include the following. First, we expect that the UTPs will obtain e-FAST ultrasound images of good quality, successfully transmit them securely, and real-time, via cellular BGAN networks to the ED. Secondly, we expect that the images obtained from the moving ambulances will be comparable to those obtained at the ED along the dimensions of quality, resolution and details. Finally, we expect the quality of the images to be similar in all cases independent of the patients’ body mass index.

## Discussion

Findings from this study will have the following important implications for patient care. First, by using a prototype portable ultrasound device, one can perform point-of-care ultrasound, and transmit the obtained data from a pre-hospital setting, real-time, to experts in the hospital, we may be able to facilitate medical care of a patient located in a remote or austere setting [27,28,30-33]. Second, the novel TS system has a potential to facilitate communication between person obtaining images and institution’s medical experts as well as real time transfer of clinical data from prehospital setting to the ED. In the manner of telemedicine, it would especially provide critical information regarding the patient’s condition that could permit for more expeditious hospital care during that golden hour and possibly reduce mortality risk. Third, findings from this study, if successful, will lead to widespread adoption of utility of ultrasound diagnosis and management of trauma patients in the pre-hospital setting. This may in turn have implications for its adoption by the military in the battlefield. Although ultrasound is a well-established technology to perform e-FAST examination in the ED setting, its use in the pre-hospital setting will enable patients to be triaged to a higher acuity level with blunt trauma. Knowing that a patient has free fluid in the abdomen even with normal vital signs (as many patients do) place patients at higher risk of mortality and morbidity from their trauma. Alerting emergency departments to see these ultrasound images will allow centers to prepare and allocate resources in preparation for patient’s arrival. This would be extremely helpful in a rural setting, where decisions about air transport versus ground transport need to be made. Finally, the ability of portable ultrasonography to provide increased local diagnostic capabilities may prevent evacuation to higher-level facilities solely for other diagnostic imaging [30,31].

## Abbreviations

eFAST: Extended – Focused Abdominal Sonography in Trauma; IRB: Institutional Review Board; QUIS: Questionnaire for User Interaction and Satisfaction; ED: Emergency Department; UTP: Ultrasound Trained Physician; HUMC: Hackensack University Medical Center; DoD: Department of Defense; DTRA: Defense Threat Reduction Agency; N: Total number of research subjects; TS: TeleSonography; BMI: Body Mass Index; MANOVA: Multiple Analysis of Variance, statistical test; BGAN: Broad Ground Area Networks.

## Competing interests

This study was supported by funding received Department of Defense Contract Funds, Grant# HDRTA1-09-C-0059 Under the Defense Threat Reduction Agency (DTRA) of the Department of Defense (PI: Joseph Feldman, MD).

## Authors’ contributions

HM, MD, MEE, BSEE, a Board Certified Emergency Medicine Physician with an extensive background in advanced technology research and development originated the idea of interfacing two “off-the-shelf” products to develop a remote medical ultrasound system and is the Principal Investigator. He identified and worked with a company in the broadcast industry to adapt their technology for medical ultrasound transmission via cellular, satellite, Wi-Fi and other pathways. He contributed significantly to the technical/engineering narrative in this project. VH, PhD, will work extensively on technical implementation, obtaining ultrasound images and transmission via live broadcast unit, as well as image analysis and evaluations. She also assisted with protocol development. Dr. Hazelwood serves as a Co-Investigator on this project and her role will include: 1) 1dentification, vetting, and experimental testing of communications and ultrasound technologies; and 2) development of these and associated technologies to attain clinical and commercial readiness in the shortest possible time frame. CO, MD, MPH, a practicing Emergency Physician and director for research in Emergency Department, was in charge of development of protocol and scientific appropriateness of entire project, as well as selection of pre-validated user satisfaction questionnaire tool, data collection and management, including study methods and design, and selection of study team. Ensuring a significant level of scientific rigor all thru the entire process. She is also responsible study review, IRB submission. Dr. Ogedegbe is responsible for the overall coordination of the study, preparation of abstracts and manuscripts from data on this project. WC, PhD, a quantitative psychologist, was the biostatiscian on the study. He was responsible for the power analysis, and the data analysis plan. JF, MD, a practicing Emergency Physician and Chair of Department of Emergency Medicine, was very involved in selection of the specific project, ensuring that specific study design and goals remained aligned with the goals of the study sponsors. Also reviewed and endorsed all aspects of manuscript creation.

## Authors’ information

Herman Morchel, MD a practicing Board Certified Emergency Medicine Physician holds Bachelors and Masters degrees in Electrical Engineering as well as two United States Patents. He has decades of experience in advanced technology research and development. Areas of concentration include electronics, computers, and communications systems. Adjunct Clinical Professor of Biomedical Engineering at Stevens Institute of Technology, Hoboken, New Jersey.

Vikki Hazelwood, PhD a Biomedical Engineering Professor currently at Stevens Institute, works part time with the team, has experience in development of biomedical devices in the lab, as well as translating them to commercial products for use in the clinic.

Chinwe Ogedegbe, MD, MPH, FACEP a practicing Board Certified Emergency Physician and Director for research in Emergency Department, member of the Institution’s IRB committee, and Associate Professor of St Georges University Department of Emergency Medicine.

William Chaplin, PhD, a quantitative psychologist, was the biostatiscian on the study. He has worked collaboratively on prior projects with our team. He works primarily in the Department of Psychology, at St. Johns University, Queens NY.

Joseph Feldman, MD, FACEP a practicing Board Certified Emergency Physician and Chair of Department of Emergency Medicine and Emergency Services at the 775-bed institution, Director of Medical Education for the St. Georges University School of Medicine program at Hackensack University Medical Center.

## Pre-publication history

The pre-publication history for this paper can be accessed here:

http://www.biomedcentral.com/1471-227X/12/19/prepub
